# The image quality and feasibility of solitary delayed [^68^ Ga]Ga-PSMA-11 PET/CT using long field-of-view scanning in patients with prostate cancer

**DOI:** 10.1186/s13550-024-01076-8

**Published:** 2024-02-06

**Authors:** Xiaofeng Yu, Lian Xu, Gang Huang, Jianjun Liu, Ruohua Chen, Yumei Chen

**Affiliations:** Department of Nuclear Medicine, Renji Hospital, School of Medicine, Shanghai Jiaotong University, No.160 Pujian Road, Pudong District, Shanghai, 200127 People’s Republic of China

**Keywords:** Long field-of-view PET/CT, [^68^ Ga]Ga-PSMA-11, Image quality, Prostate cancer, Solitary delayed imaging

## Abstract

**Background:**

Previous studies have demonstrated that delayed [^68^ Ga]Ga-PSMA PET/CT imaging improves lesion detection compared to early [^68^ Ga]Ga-PSMA PET/CT in patients with prostate cancer. However, the sole use of delayed [^68^ Ga]Ga-PSMA PET/CT has been limited due to the insufficient number of photons obtained with standard PET/CT scanners. The combination of early and delayed [^68^ Ga]Ga-PSMA standard PET/CT may be considered, and it is challenging to incorporate into a high-demand clinical setting. Long field-of-view (LFOV) PET/CT scanners have higher sensitivity compared to standard PET/CT. However, it remains unknown whether the image quality of solitary delayed [^68^ Ga]Ga-PSMA LFOV PET/CT imaging is adequate to satisfy clinical diagnostic requirements. Therefore, the purpose of this study was to evaluate the image quality of delayed [^68^ Ga]Ga-PSMA LFOV PET/CT and examine the feasibility of utilizing delayed [^68^ Ga]Ga-PSMA LFOV PET/CT imaging alone in patients with prostate cancer.

**Methods:**

The study sample consisted of 56 prostate cancer patients who underwent [^68^ Ga]Ga-PSMA-11 LFOV PET/CT scanning between December 2020 and July 2021. All patients were subjected to early LFOV PET/CT imaging at 1-h post-injection as well as delayed LFOV PET/CT imaging at 3-h post-injection using [^68^ Ga]Ga-PSMA-11. The image quality and diagnostic efficiency of solitary delayed [^68^ Ga]Ga-PSMA-11 LFOV PET/CT imaging was analyzed.

**Results:**

The results showed that delayed [^68^ Ga]Ga-PSMA-11 LFOV PET/CT yielded satisfactory image quality that fulfilled clinical diagnostic benchmarks. Compared to early imaging, delayed [^68^ Ga]Ga-PSMA-11 LFOV PET/CT demonstrated heightened lesion SUVmax values (11.0 [2.3–193.6] vs. 7.0 [2.0–124.3], *P* < 0.001) and superior tumor-to-background ratios (3.3 [0.5–62.2] vs. 1.7 [0.3–30.7], *P* < 0.001). Additionally, delayed [^68^ Ga]Ga-PSMA-11 LFOV PET/CT detected supplementary lesions in 14 patients (25%) compared to early imaging, resulting in modifications to disease staging and management plans.

**Conclusions:**

In summary, the findings indicate that the image quality of delayed [^68^ Ga]Ga-PSMA-11 LFOV PET/CT is satisfactory for meeting clinical diagnostic prerequisites. The use of solitary delayed [^68^ Ga]Ga-PSMA-11 LFOV PET/CT imaging in prostate cancer simplifies the examination protocol and improves patient compliance, compared to [^68^ Ga]Ga-PSMA-11 standard PET/CT which necessitates both early and delayed imaging.

## Introduction

Prostate cancer (PCa) ranks as the second most prevalent cancer in men and constitutes a significant contributor to global cancer mortality [1]. The implementation of [^68^ Ga]Ga-PSMA positron emission tomography/computed tomography (PET/CT) is critical for initial staging and biochemical recurrence (BCR) for the localization of prostate cancer lesions [2–7]. While early [^68^ Ga]Ga-PSMA PET/CT is performed at 60 min post-injection (p.i.) of radiotracer [8, 9], later image acquisition could detect more lesions and clarify ambiguous findings due to the increase in lesion uptake and an improved target-to-background ratio over time [10–12]. The time-activity curves (TACs) of the prostate lesion, lymph node, and bone metastases demonstrate a steady increase over the initial 60 min p.i., while the lesion uptake values at the delayed imaging point (180 min p.i) surpass the respective values at 60 min p.i. [13].

Despite the advantages associated with delayed acquisition, certain factors present challenges to conducting [^68^ Ga]Ga-PSMA PET/CT imaging solitarily at delayed time points. These include the short half-life of Gallium-68 and the limited sensitivity of standard PET/CT. While the combination of early and delayed imaging may be considered, it is challenging to incorporate into a high-demand clinical setting. Moreover, patient compliance may be suboptimal when required to undergo concurrent early and late imaging. This issue, however, might be mitigated with the advent of the LFOV PET/CT scanner. This scanner enhances sensitivity by up to 40 times compared to standard PET/CT [14]. By extending the geometric coverage to envelop the entire body, LFOV PET/CT heralds a significant leap in sensitivity. Recent studies have underscored the advantages of LFOV PET/CT over standard PET/CT in [^18^F]F- FDG. However, whether the image quality of delayed imaging utilizing [^68^ Ga]Ga-PSMA-11 LFOV PET/CT is robust enough to meet clinical diagnostic needs and whether a solitary delayed [^68^ Ga]Ga-PSMA PET/CT acquisition is feasible with LFOV PET/CT in PCa have yet to be assessed.

In this study, we evaluate the image quality of delayed [^68^ Ga]Ga-PSMA-11 LFOV PET/CT and examine the feasibility of utilizing delayed [^68^ Ga]Ga-PSMA-11 LFOV PET/CT imaging alone in patients with prostate cancer.

## Materials and methods

### Participants

**Fig. 1:**
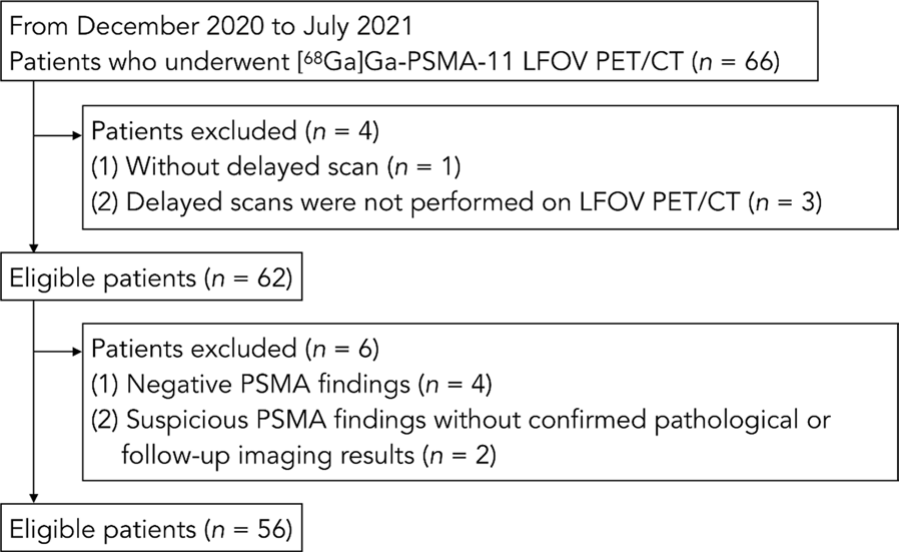
Flowchart of the 56 patient enrollment in the study

This retrospective study encompassed 56 patients with prostate cancer, who sought early and delayed [^68^ Ga]Ga-PSMA-11 LFOV PET/CT in Shanghai Renji hospital between December 2020 and July 2021 (Fig. 1). Eligibility criteria included (a) pathological confirmation of primary PCa staging or evidence of biochemical recurrence (BCR) with a PSA value exceeding 0.2 ng/ml; (b) positive [^68^ Ga]Ga-PSMA-11 PET/CT findings [15]; (c) histological or follow-up imaging confirmation of all lymph node metastases (LNM); (d) verification of bone and solid organ metastases through conventional imaging (CT, MRI and bone scan) or follow-up PET/CT. The study was approved by the Institutional Review Board of Renji Hospital affiliated to Shanghai JiaoTong University and conducted in line with the Helsinki Declaration. Written informed consents were obtained from all participants.

### PET/CT acquisition and imaging reconstruction

Both early and delayed imaging were performed on all patients using the LFOV PET/CT system (uEXPLORER, United Imaging Healthcare, Shanghai, China). Adhering to the guidelines [16], early and delayed imaging were conducted at 1-h post-injection (p.i.) and 3-h p.i., respectively. All patients received 40 mg of orally administered furosemide and 1 L of water for hydration, followed by early [^68^ Ga]Ga-PSMA-11 PET/CT scans. A CT scan, equipped with a fixed tube voltage of 120 kV and a dose modulation technique to automatically adjust mAs, was carried out prior to the early PET scan. The LFOV PET scan, encompassing the entire human body in one bed position, was performed with an acquisition time of 5 min for both early and delayed imaging.

Standard ordered subset expectation maximization algorithm was applied for PET reconstruction. Appropriate corrections including decay, scatter, random, dead time, attenuation and normalization were applied to the reconstructions.

### Image analyses

All the PET images were evaluated by two nuclear medicine physicians (with more than 10 years of PET/CT experience) on a dedicated workstation (uWS-MI, United Imaging Healthcare, Shanghai, China). These readers were blinded to patient demographics and the imaging time points. They independently scored the images using a 5-point Likert scale to reflect an overall assessment of image quality, rating them as (1) poor, (2) acceptable, (3) fair, (4) good, or (5) excellent. In the event of a discrepancy, a third, more experienced nuclear medicine specialist (with 20 years of PET/CT experience) provided a deciding vote. The liver signal-to-noise ratio (SNR) served as an additional parameter to assess image quality, calculated as the quotient of the liver’s mean standardized uptake value (SUVmean) and its standard deviation (SD).

For patients with PCa who underwent LFOV PET/CT, the PSMA-avid lesion uptake value was determined in terms of the maximum standardized uptake value (SUVmax) on both early and delayed images. Physiological [^68^ Ga]Ga-PSMA-11 uptake values in background regions (including the liver, spleen, blood pool, parotid gland, gluteus maximus, and bladder) were gauged via SUVmean in both early and delayed images. The tumor-to-background ratio (TBR) was computed by dividing the lesion SUVmax by the liver SUVmean. Furthermore, based on varying anatomical locations, all PSMA-avid lesions were classified into one of the following categories: primary prostate lesions or local recurrence, lymph node metastases (LNM), bone metastases, and solid organ metastases. All LNMs were further subclassified according to diameter (small LNM with a diameter < 10 mm, large LNM ≥ 10 mm) and location (pelvic or extra-pelvic). The SUVmax of all PSMA-avid lesions on delayed images were compared to those on early images, with the SUVmax change calculated as [(delayed SUVmax—early SUVmax)/early SUVmax]. The difference and change in SUVmax between small and large LNM were further compared.

### Statistical analysis

Dedicated statistical analysis was used for analysis (SPSS Statistics, version 25.0 IBM Corp., Armonk, NY). Shapiro–Wilk test and Kolmogorov–Smirnov test were used to test the normality of parameters in the quantitative analysis. For normal distributed variables, mean and SD were given. For non-normal distributed variables, median and range were given. A student’s t test was used to compare qualitative scores, liver SNR and SUV_mean_ values of background regions. A Wilcoxon matched-pairs signed rank test was performed to compare SUV_max_ values of PSMA-avid lesions and TBR between the two scans. A Mann–Whitney test was used to compare the quantitative parameters for subgroup analysis on lymph nodes. A *p* value < 0.05 was considered statistically significant.

## Results

### Study population

The characteristics of the patients are delineated in Table 1. The cohort consisted of 56 patients diagnosed with prostate cancer (36 primary, 20 BCR) who underwent [^68^ Ga]Ga-PSMA-11 LFOV PET/CT imaging at two distinct time points: 1-h p.i. and 3-h p.i.. Each patient presented with PSMA-avid prostate cancer lesions, with a total of 226 pathological lesions identified and subjected to analysis.

**Table 1:** Demography and clinical characteristics of the 56 enrolled patients

Characteristic	Value
*Indication for PET/CT*	
Initial staging	36^†^
Recurrence detection	20^†^
Age (year)	68.7 ± 7.1
[^68^ Ga]Ga-PSMA dose (MBq)	138.6 ± 18.6
*Time point p.i. (min)*	
Early imaging	59.0 ± 9.8
Delayed imaging	181.2 ± 21.5
*PSA level (ng/mL)*	
Primary	30.7 (1.7 ~ 2301.0)
BCR	0.9 (0.3 ~ 17.0)
*Gleason score (GS)*	
3 + 4	11^†^
4 + 3	17^†^
4 + 4	17^†^
3 + 5	1^†^
5 + 3	1^†^
4 + 5	7^†^
5 + 5	1^†^
NA^※^	1^†※^

Data are presented as means ± standard deviations or median (range)

^†^Indicates number of patients

^※^The pathological diagnosis of the one patient was prostate cancer, while lacking GS value

### Image quality analyses

On the delayed LFOV PET/CT imaging, 16 patients were assessed as 5 points, 32 patients were assessed as 4 points and eight patients were assessed as 3 points, and none of patients were assessed as 2 or 1 points. The image quality on the delayed LFOV PET/CT imaging in all patients met clinical diagnostic needs.

**Fig. 2:**
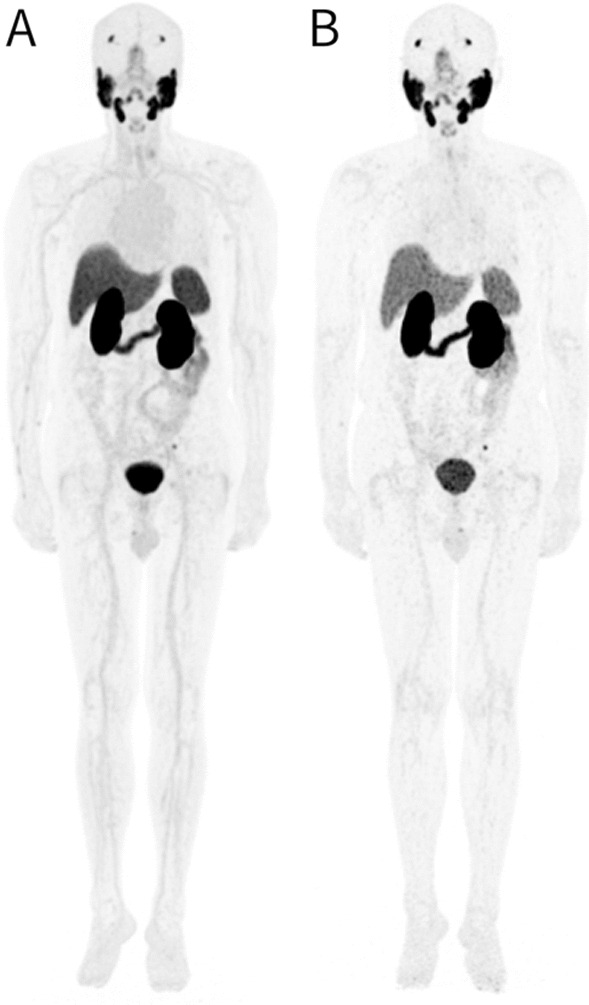
An exemplary instance of the maximum intensity projection (MIP) on early and delayed LFOV PET/CT was shown (tiles A and B). The image quality of A and B were evaluated as 5 and 4 points, respectively. The liver SNR were 19.3 and 8.9, respectively

The image quality on LFOV PET/CT was mildly reduced compared to the early image (4.1 ± 0.6 at 3 h p.i. vs. 4.9 ± 0.4 at 1 h p.i., *p* < 0.001). The SNR of delayed image on LFOV PET/CT was lower than that in early image (10.0 ± 2.2 at 3 h p.i. vs. 15.6 ± 4.0 at 1 h p.i., *p* < 0.001) (Fig. 2).

For 56 PCa patients performed on LFOV PET/CT, the delayed images exhibited a significant decrease in SUVmean across the majority of the background regions, including the liver, spleen, blood pool, gluteus maximus, and bladder (Table 2). Notably, the diminished uptake in the bladder facilitated superior delineation of the adjacent lesions, as evidenced by the comparison of SUVmean in bladder at different time intervals (7.4 ± 2.6 at 3 h p.i. vs. 28.7 ± 24.5 at 1 h p.i., *p* < 0.001). For all PSMA-avid lesions, the delayed images demonstrated significantly higher SUV_max_ values (11.0 [2.3 ~ 193.6] vs. 7.0 [2.0 ~ 124.3], *p* < 0.001) and increased TBR (3.3 [0.5 ~ 62.2] vs. 1.7 [0.3 ~ 30.7], *p* < 0.001) in comparison with the early images (Table 3).

**Table 2:** Comparison of background SUV_mean_ between the early and delayed images

Background regions	Early	Delayed	*p*
Liver	4.5 ± 1.4	3.5 ± 1.3	< 0.001
Spleen	8.4 ± 2.6	5.5 ± 2.0	< 0.001
Blood pool	1.3 ± 0.3	0.7 ± 0.2	< 0.001
Parotid gland	11.0 ± 2.9	13.2 ± 4.0	< 0.001
Muscle	0.4 ± 0.1	0.2 ± 0.1	< 0.001
Bladder	28.7 ± 24.5	7.4 ± 2.6	< 0.001

Data are presented as means ± standard deviations

SUV_mean_ = mean standard uptake value

**Table 3:** Comparison of SUV_max_ and TBR between the early and delayed images

Type & site	No. of lesions	SUV_max_				TBR			
		Early	Delayed	*p*	Change (%)	Early	Delayed	*p*	Change (%)
Primary	49*	13.4 (3.5 ~ 89.6)	21.8 (2.8 ~ 153.0)	< 0.001	48.2 (− 20.0 ~ 134.2)	3.2 (1.1 ~ 26.1)	6.9 (1.5 ~ 52.9)	< 0.001	85.5 (34.8 ~ 198.7)
Local recurrence	5*	13.5 (10.9 ~ 22.4)	24.1 (16.5 ~ 35.3)	0.0625	69.8 (52.0 ~ 116.3)	3.3 (2.1 ~ 4.7)	6.7 (3.1 ~ 8.7)	0.0625	96.7 (51.7 ~ 153.7)
LNM	98	5.9 (2.0 ~ 124.3)	10.0 (2.3 ~ 193.6)	< 0.001	66.5 (− 24.0 ~ 249.0)	1.4 (0.3 ~ 30.7)	2.8 (0.5 ~ 62.2)	< 0.001	108.4 (− 7.0 ~ 304.2)
Bone	63	5.7 (2.1 ~ 82.6)	7.9 (3.0 ~ 122.2)	< 0.001	41.5 (− 57.0 ~ 230.9)	1.4 (0.6 ~ 22.6)	2.8 (0.6 ~ 37.8)	< 0.001	91.2 (− 42.0 ~ 278.2)
Solid organ	3	5.1 (4.4 ~ 18.0)	5.0 (4.6 ~ 29.8)	0.500	15.6 (− 9.8 ~ 65.5)	1.6 (1.2 ~ 4.4)	2.5 (1.6 ~ 9.8)	0.250	52.0 (34.4 ~ 124.5)

Data are presented as median (range)

SUV_max_ = maximum standard uptake value, TBR = target-to-background ratio

*The total number of primary prostate lesions and local recurrence lesions was 52 and 10, respectively, whereas three primary and five local recurrence lesions could not detect SUV_max_ value and TBR in early images owing to the urinary bladder interference

### Lesion detectability

#### Patient-based analysis

Of the 56 patients, 14 patients (25.0%) exhibited additional PSMA-avid lesions in the delayed imaging in comparison to the early images. Within this subset, the additional findings in eight patients (57.1%) had a significant impact on disease staging and subsequent clinical management strategies. Specifically, additional lesions within the prostate region were detected in five of these eight patients, while one patient presented with bone metastasis, another with LNM, and the last patient showed both prostate region lesion and LNM (Table 4).

**Table 4:** Summary of the eight patients with the disease upstaging in the delayed images

Patient no	PSA	GS	Type	Site	Early SUV_max_	Delayed SUV_max_	Disease upstaging
1	32.3	8	Primary	Seminal vesicle	4.8	8.1	T_2_N_1_M_0_ → T_3_N_1_M_0_
2	24.0	7	Primary	Prostate gland	NA	9.6	T_X_N_0_M_0_ → T_3_N_0_M_0_
				Seminal vesicle	1.7	15.0	
3	17.0	8	BCR	Local recurrence	NA	25.8	LR_X_N_0_M_1b_ → LR_1_N_1_M_1b_
				Pelvic LNM	3.6	6.1	
4	1.3	8	BCR	Local recurrence	NA	14.1	LR_X_N_1_M_0_ → LR_1_N_1_M_0_
5	0.8	8	BCR	Local recurrence	NA	13.6	LR_X_N_0_M_0_ → LR_1_N_0_M_0_
6	0.6	7	BCR	Local recurrence	NA	8.1	LR_X_N_0_M_0_ → LR_1_N_0_M_0_
7	1.0	8	BCR	Pelvic LNM	NA	15.2	LR_X_N_0_M_0_ → LR_0_N_1_M_1a_
				Extra-pelvic LNM	3.3	6.9	
				Extra-pelvic LNM	3.3	5.3	
8	0.3	8	BCR	Bone	2.2	7.3	LR_X_N_0_M_0_ → LR_0_N_0_M_1b_

SUV_max_ = maximum standard uptake value

NA = not applicable due to the high radioactivity signal within the bladder

LR_X_ = unknown status of local recurrence owing to the interference from the bladder

LR_1_ = local recurrence

#### Lesion-based analysis

A total of 226 PSMA-avid lesions were identified in the 56 patients, including 62 PSMA-avid lesions in prostate region (52 primary lesion, ten local recurrence), 98 LNM (79 pelvic, 19 extra-pelvic), 63 bone metastases, and three solid organ metastases (two lung, one liver). Notably, 22 lesions (9.7%) were solitarily identified in the delayed images, comprising three primary lesions (1.3%), five local recurrences (2.2%), one bone metastasis (0.4%), and 13 LNM (5.8%). Among these 22 additional lesions, 11 (50.0%) led to patient upstaging and alterations in management plans (Table 4).

**Fig. 3:**
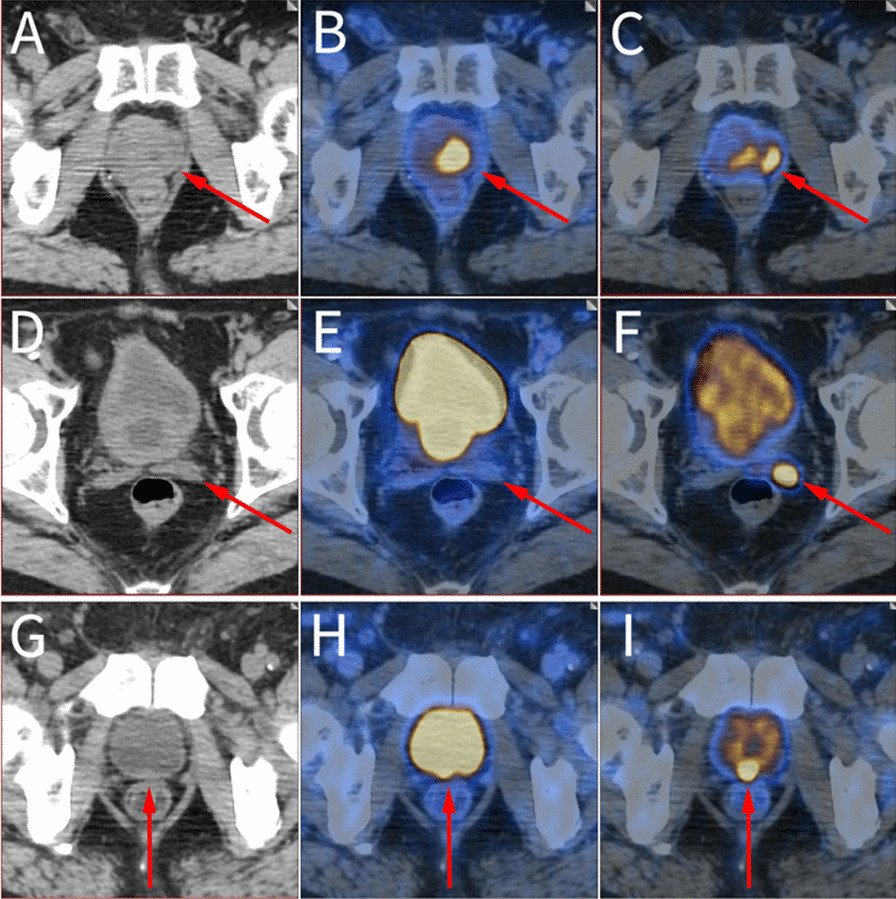
Delayed images showed additional lesion detection in prostate region. [^68^ Ga]Ga-PSMA LFOV PET/CT in a 78-year-old primary PCa patient after TURP (tiles **A**–**F**). The high radioactivity within the bladder and enlarged prostatic urethra impeded the detection of PSMA-avid lesions in early images (tiles **B** and **E**). A locally lesion at the left lobe of prostate gland was discernible in delayed image, with a SUV_max_ value of 9.6 (tile **C**). In addition, late scan detected invasion of left seminal vesicle (tile **F**), which was not distinguishable in the early scan, the SUV_max_ value was 1.7 in the early image, while the delayed image showed a SUV_max_ value up to 15.0. [^68^ Ga]Ga-PSMA LFOV PET/CT in a 70-year-old BCR patient (tiles **G**–**I**). A locally recurrent lesion at the prostate bed was detected at the delayed images, with a SUV_max_ value of 25.8 (tile **I**), which was unclear in early image due to the high radioactivity within the bladder (tile **H**)

The eight additional lesions in prostate region included five lesions in five BCR patients and three lesions in two primary PCa patients. The diminished radioactivity within the urinary bladder in the delayed images facilitated the clarification of ambiguous findings or the detection of additional lesions. One primary PCa patient was able to clarify an ambiguous lesion in the seminal vesicle through delayed imaging (Table 4. Patient no. 1). The SUV_max_ value of the lesion was 4.8 on early images. Due to the interference of high radioactivity within the bladder, the initial diagnosis was inconclusive in the early images. Delayed images revealed an increased SUVmax value of 8.1, aiding in the identification of this lesion as metastasis. Another primary PCa patient, who had undergone transurethral resection of the prostate (TURP), detected additional lesions in both the prostate and seminal vesicle (Table 4. Patient no. 2, Fig. 3. tiles A—F). A typical BCR patient identified a locally recurrent lesion at the prostate bed in the delayed PET/CT imaging (Table 4. Patient no. 3, Fig. 3. tiles G—I). Seven of the eight additional lesions resulted in disease upstaging and changes in the management plan (Table 4. Patient no.1–6).

**Fig. 4:**
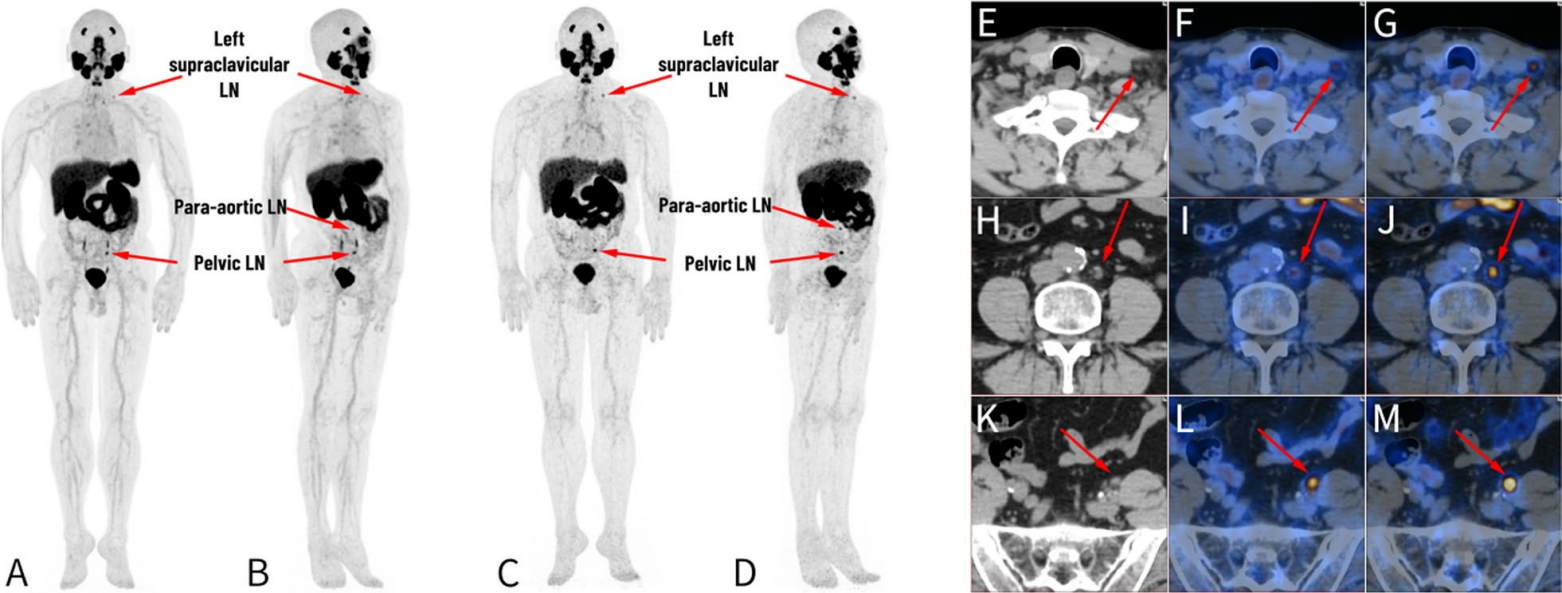
Delayed images on LFOV PET/CT showed the additional LNM in a 62-year-old primary PCa patient. Left supraclavicular LNM, para-aortic LNM and pelvic LNM could be detected on MIP of delayed LFOV images (tiles **C**–**D**), whereas MIP of early images could not make the clinically sufficient diagnoses (tiles **A**–**B**). Left supraclavicular small LNM (tiles **C**–**D**, **G**) was more distinguishable in the delayed image due to the increased SUV_max_ value (3.3 at 1 h p.i. vs. 5.3 at 3 h p.i.), which was confirmed as metastasis by LN biopsy. The para-aortic small LNM was faintly visible in the early image (tiles **B**, **I**), while it showed the increased SUV_max_ value (3.3 at 1 h p.i. vs. 11.1 at 3 h p.i.) in the delayed image (tiles **D**, **J**). Multiple foci with avid [^68^ Ga]Ga-PSMA activity were demonstrated along the bilateral urinary tracts in early images (tiles **A**, **B**, **L**), whereas only one intense [^68^ Ga]Ga-PSMA activity was observed in the left pelvic region in delayed images, with a SUVmax value of 15.2 (tiles **C**, **D**, **M**), the CT image showed a small nodular lesion with a size of 7.5*5.5 mm, which was verified as LNM. Without the urinary urine activity interference, the pelvic LNM was more visible on delayed image

In terms of the locations of 13 additionally detected LNM in the delayed [^68^ Ga]Ga-PSMA-11 LFOV PET/CT (98 at 3 h p.i. vs. 85 at 1 h p.i.), eight (61.5%) were in the pelvic region, and the remaining five (38.5%) were in the extra-pelvic region. The LFOV PET/CT proved advantageous for the detection of PSMA-avid lesions in the extra-pelvic region (Fig. 4. tiles E—J). The prompt localization of PSMA-avid lesions facilitated disease upstaging and treatment modifications (Table 4. Patient no.7).

**Fig. 5:**
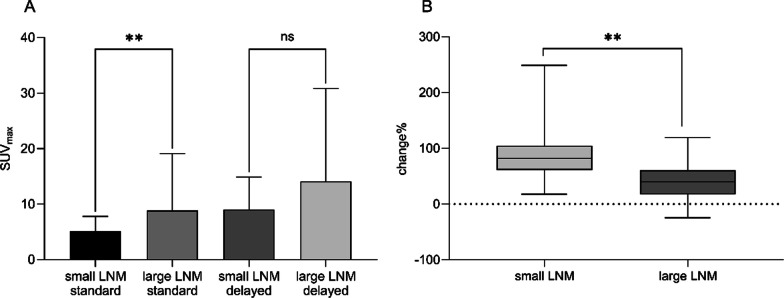
Delayed images on tLFOV PET/CT showed higher benefits to small LNM. **A** Comparison of the SUV_max_ values of small LNM and large LNM, respectively, for the early and delayed images. The difference between small and large LNM on the early images was statistically significant (*p* = 0.0004), with a SUV_max_ value of 5.1(2.0 ~ 54.1) and 8.8(2.8 ~ 124.3), respectively, while no statistical difference was found on the delayed images (*p* = 0.0719), with a SUV_max_ value of 9.0(2.8 ~ 109.7) and 14.1(2.3 ~ 193.6), respectively. **B** The uptake change of small LNM and large LNM on the early and delayed images. The uptake change in small and large LNM was increased by 81.7% and 40.0%, respectively (*p* < 0.001)

In terms of the sizes of the 13 additional LNM detected in the delayed scans, all were classified as small LNM (diameter less than 10 mm), constituting 19.7% of all detected small LNM. The SUVmax of all small LNM was 5.1 (2.0 ~ 54.1) on early images, which significantly elevated to 9.0 (2.8 ~ 109.7) on delayed images (*p* < 0.001). Furthermore, the TBR of all small LNM was 1.3 (0.3 ~ 11.2) and 2.8 (0.5 ~ 31.1), respectively, on early and delayed images (*p* < 0.001). The elevated lesion SUVmax value and enhanced TBR in the delayed images contributed to an increased detection rate of small LNM via the ultra-sensitive LFOV PET/CT. A significant difference in SUVmax was observed between small and large LNM on the early images (*p* < 0.001), while no difference was discerned on the delayed images (*p* = 0.072). The SUVmax change in small LNM increased by 81.7%, whereas large LNM increased by 40.0% (Fig. 5). The small LNM derived greater benefits from the delayed images compared to large LNM, particularly those adjacent to the urinary ureter and bladder, where the delayed images may aid in clarifying these lesions. A representative case demonstrated that a small LNM near the left ureter was clarified in the delayed images (Table 4. Patient no. 7, Fig. 4. tiles K—M).

**Fig. 6:**
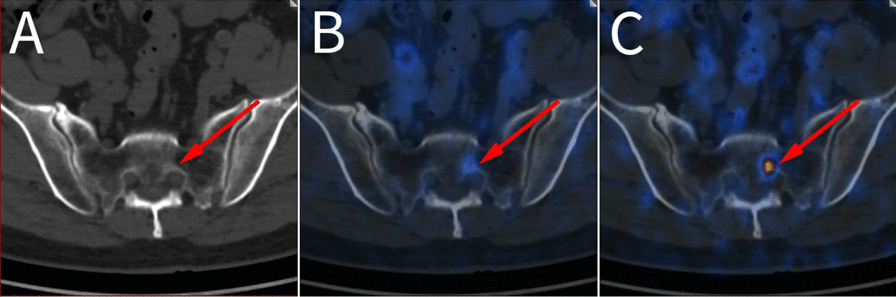
Delayed images on LFOV PET/CT clarified the unclear finding in sacrum in a 73-year-old BCR patient. The sacral metastasis was vaguely visible at the early image (tile **B**), with a SUV_max_ value of 2.2. Delayed image showed the SUV_max_ value increased to 7.3 (tile **C**). The CT image showed no clear morphological change (tile **A**)

With regard to bone metastases, the delayed image facilitated the clarification of an ambiguous finding in the sacrum. The early image exhibited a marginally focal [^68^ Ga]Ga-PSMA uptake value of 2.2. However, the SUV_max_ value increased to 7.3 in the delayed image (Fig. 6). The persistent increase in this bone metastasis observed in the delayed imaging led to the upstaging of this patient, thereby necessitating a modification in the management plan (Table 4. Patient no.8).

## Discussion

Previous studies have demonstrated that delayed [^68^ Ga]Ga-PSMA PET/CT imaging improves lesion detection compared to early [^68^ Ga]Ga-PSMA PET/CT in patients with prostate cancer. However, the solitary use of delayed [^68^ Ga]Ga-PSMA PET/CT has been limited due to the insufficient number of photons obtained with standard PET/CT scanners. The combination of early and delayed [^68^ Ga]Ga-PSMA standard PET/CT may be considered, and it is challenging to incorporate into a high-demand clinical setting. LFOV PET/CT scanners have higher sensitivity compared to standard PET/CT. However, it remains unknown whether the image quality of solitary delayed [^68^ Ga]Ga-PSMA LFOV PET/CT imaging is adequate to satisfy clinical diagnostic requirements. To the best of our knowledge, this study represents the first application of [^68^ Ga]Ga-PSMA-11 LFOV PET/CT to evaluate the image quality of delayed image and to scrutinize the feasibility of solitary delayed [^68^ Ga]Ga-PSMA-11 LFOV PET/CT in PCa.

Our study demonstrated that the image quality of delayed imaging utilizing [^68^ Ga]Ga-PSMA-11 LFOV PET/CT is sufficiently robust to meet clinical diagnostic requirements. Furthermore, the delayed [^68^ Ga]Ga-PSMA-11 LFOV PET/CT exhibited higher lesion SUVmax and TBR (*p* < 0.001) compared to early image in the prostate region, LNM, and bone metastases.

Focal retained activity in the bladder and ureter is a frequent clinical challenge, which complicates the detection of lesions in the prostate region [17]. Previous studies have shown that the combination of early and delayed [^68^ Ga]Ga-PSMA PET/CT scans can enhance lesion detectability, especially in BCR patients [16, 18–20]. Furosemide administration with abundant hydration could further delineate local recurrence lesions [3]. However, due to the short half-life of Gallium-68, the image quality of delayed image in standard PET/CT is poor with a low counting rate. Therefore, the solitary use of delayed imaging in standard PET/CT was deemed unfeasible. While the combination of early and delayed imaging could be considered, its integration into a clinical setting presents challenges due to the demanding nature of clinical work. Furthermore, patient compliance may be less than ideal when required to undergo simultaneous early and late imaging. The LFOV PET/CT scanner, which boasts a sensitivity up to 40-fold higher compared to the standard PET/CT [21–26], is ideally suited to address this issue. Our study demonstrated that [^68^ Ga]Ga-PSMA-11 LFOV PET/CT could enhance image interpretation when combined with forced diuresis and delayed imaging. Owing to the significant tracer reduction in the background regions, particularly in the bladder, superior delineation of the prostate lesion could be achieved. This is especially beneficial for the detection of local relapse and adjacent LNM.

Early [^68^ Ga]Ga-PSMA PET/CT is acquired with a duration of 3–5 min per bed position in standard PET/CT. This could result in a total of up to 20–30 min for early imaging, and potentially two to three times longer for delayed imaging due to the limited sensitivity. Delayed imaging in standard PET/CT is typically performed limited to the pelvic region to reduce scan time; this may result in missed PSMA-avid lesions in the extra-pelvic region. The ultra-high-sensitive LFOV PET/CT may only necessitate an acquisition time of 30–45 s to provide an image quality equivalent to that of standard PET/CT with extended acquisition settings in [^18^F]F-FDG [23, 27, 28]. In our study, we adopted a 5-min acquisition time for both early and delayed [^68^ Ga]Ga-PSMA-11 PET/CT imaging using LFOV PET/CT, while still providing satisfactory image quality to meet clinical requirements. The LFOV PET/CT scanner can encompass the entire human body in a solitary bed position, facilitating lesion detection in both pelvic and extra-pelvic regions. In summary, our study demonstrated that delayed acquisition on [^68^ Ga]Ga-PSMA-11 LFOV PET/CT could detect extra-pelvic metastases with only a 5-min acquisition time, thereby influencing patient staging and management plans.

Although our previous study demonstrated that a combination of early dynamic [^68^ Ga]Ga-PSMA LFOV PET/CT imaging during the initial 6 min and the early imaging at 1 h p.i. could better delineate pathological lesions, it could potentially overlook lesions smaller than 10 mm due to the low counting rate in the early stage [13]. The current study found that small LNM derived greater benefits from the delayed [^68^ Ga]Ga-PSMA LFOV PET/CT compared to large LNM, owing to a significant increase in the SUVmax (*p* < 0.001). Moreover, all the additional LNM detected in the delayed image were small. Consequently, the current study substantiated that the delayed [^68^ Ga]Ga-PSMA LFOV PET/CT enhanced the detection rate of these small lesions, owing to its high sensitivity and resolution.

Our study was subject to several limitations. Firstly, it was a solitary-center retrospective study, and the patient sample size was relatively small. Secondly, while our study assessed the image quality and lesion detection rate on [^68^ Ga]Ga-PSMA-11 LFOV PET/CT, there are numerous other mature PSMA radiotracers widely available, such as [^18^F]F-PSMA tracers, it may demonstrate better diagnostic value with enough counts in delayed imaging due to its longer half lives, whether our result is applicable to other PSMA radiotracers still needs further investigation.

## Conclusions

The image quality of delayed [^68^ Ga]Ga-PSMA-11 LFOV PET/CT is satisfactory for meeting clinical diagnostic prerequisites. The use of solitary delayed [^68^ Ga]Ga-PSMA-11 LFOV PET/CT imaging in prostate cancer simplifies the examination protocol and improves patient compliance, compared to [^68^ Ga]Ga-PSMA-11 standard PET/CT which necessitates both early and delayed imaging.

## Data Availability

All data and material are available upon request from the corresponding author.
